# Supplementary material for: Severe leptospirosis in tropical and non-tropical areas: A comparison of two french, multicentre, retrospective cohorts
Source: PLoS Negl Trop Dis. 2024 Apr 10;18(4):e0012084. doi: 10.1371/journal.pntd.0012084 (PMC11034666; doi:10.1371/journal.pntd.0012084)
Supplement: S1 Text — (DOCX) [file pntd.0012084.s001.docx]

**ADDITIONAL FILE**

**Severe Leptospirosis in Tropical and Non-Tropical Areas: A Comparison of Two French, Multicentre, Retrospective Cohorts**

.

**METHODS**

**Sources of the study data**

***Study in Réunion (two ICUs, January 2004–January 2015)***

Consecutive patients admitted to either of the two ICUs on the island for documented severe leptospirosis were included. The two ICUs were located in the Félix Guyon University Hospital and the Saint-Benoît Hospital, respectively, and were managed by the same medical team. The patients were identified by searching the electronic databases of the two hospitals.

Documentation of leptospirosis was by polymerase chain reaction for 23S ribosomal RNA on blood and/or urine and/or by a serological test (microscopic agglutination test [MAT] and/or enzyme-linked immunosorbent assay [ELISA]) showing a titre above 1:400.

The following baseline data were collected: age, sex, occupation, hobbies, exposure to animals, smoking status, alcohol use, and comorbidities (hypertension, diabetes, chronic renal failure, cirrhosis of the liver, chronic respiratory failure, ischaemic heart disease, heart failure, cancer, immunosuppression, and neurologic disease). Clinical data included fever, myalgia, arthralgia, jaundice, nausea, vomiting, abdominal pain, enlargement of the liver and/or spleen, urinary output decrease to less than 500 mL/day, haemorrhagic syndrome (at least one of the following: haemoptysis, gastrointestinal bleeding, purpura, conjunctival bleeding, haematuria, epistaxis), dyspnoea, cough, cardiovascular collapse, and meningeal syndrome. The Simplified Acute Physiology Score version II (SAPSII) and Sequential Organ Failure Assessment (SOFA) score were determined based on the most abnormal values recorded within 24 hours after admission. All patients were screened for manifestations of leptospirosis including myocarditis, pericarditis, cardiac arrest, atrial fibrillation, cardiogenic shock, acute renal failure, meningitis, encephalitis, intracranial haemorrhage, intra-alveolar haemorrhage, moderate-to severe acute respiratory distress syndrome (ARDS), liver function impairment, and gastrointestinal bleeding. The results of routine biological tests done on admission then on days 1, 2, 3, and 7 were collected, as well as the SOFA sub-scores determined at the same time points. The following therapeutic data were recorded: type and duration of antibiotic therapy; use of non-invasive ventilation (NIV) and/or invasive mechanical ventilation (MV), extra-corporeal membrane oxygenation, high-frequency oscillatory ventilation, prone positioning, and nitric oxide; renal replacement therapy (performed only by continuous veno-venous hemofiltration in this ICU) with the duration in days; vasoactive drugs; and blood transfusion.

***Study in mainland France (79 ICUs, January 2012–September 2016)***

Adults (≥18 years of age) with documented leptospirosis were identified by searching the hospital databases for code A27 (A27.0, A27.9 et A27.9) in the International Classification of Diseases-10^th^ revision. Among these patients, those who required ICU admission for severe leptospirosis were then selected. For each patient thus identified, the study investigator at each centre reviewed the medical files and extracted the data required to complete the case-report form, which also had space for recording data that were not specifically required but seemed of interest. The recorded data included chronic comorbidities, alcohol and tobacco use, history of the acute illness and clinical manifestations on ICU admission, values of the SAPS II and clinical parameters, Glasgow Coma Scale score, life-sustaining interventions, and antibiotic score.

**Definitions**

The current study compared only those variables whose definitions were identical in the two cohorts. These variables were as follows.

- Chronic comorbidities:
- cancer and/or immune deficiency defined as proven cancer within the past 5 years or radiotherapy or chemotherapy within the past 6 months or HIV infection or congenital immunodeficiency;
- diabetes mellitus type 1 or 2, with or without complications;
- history of liver cirrhosis with or without complications;
- heart failure with dyspnoea at rest or upon exertion;
- respiratory disease with dyspnoea at rest or upon exertion or with chronic hypercapnia or with continuous oxygen therapy;
- chronic kidney disease with or without a requirement for dialysis;
- chronic alcohol consumption;
- chronic tobacco consumption.
- Simplified Acute Physiology Score (SAPS II) recorded in the medical file; in France, the SAPS II is recorded routinely within 24 hours of ICU admission.
- Sequential Organ Failure Assessment (SOFA) sub-scores and total score determined from the worst clinical and laboratory values recorded on admission then daily during the first ICU week
- Clinical manifestations on ICU admission: fever, myalgia, arthralgia, meningeal syndrome; jaundice; diarrhoea, abdominal pain; dyspnoea, cough, haemoptysis
- Laboratory data: worst value within 24 hours after ICU admission for lactate (mmol/L), bilirubin (µmol/L), alanine aminotransferase (IU/L), aspartate aminotransferase (IU/L), haemoglobin (Giga/dL), platelets (Giga/L), leucocytes (Giga/L), prothrombin time, creatinine (µmol/L), urea (mmol/L), potassium (mmol/L), creatine kinase (IU/L), C-reactive protein (mg/L)
- organ-support interventions and outcomes: vasoactive drugs with the duration; invasive mechanical ventilation and/or non-invasive ventilation and/or extracorporeal membrane oxygenation, with the durations; renal replacement therapy with the duration; ICU and hospital stay lengths; in-ICU and in-hospital death.
