# Supplementary figures and images for: Severe leptospirosis in tropical and non-tropical areas: A comparison of two french, multicentre, retrospective cohorts
Source: PLoS Negl Trop Dis. 2024 Apr 10;18(4):e0012084. doi: 10.1371/journal.pntd.0012084 (PMC11034666; doi:10.1371/journal.pntd.0012084)

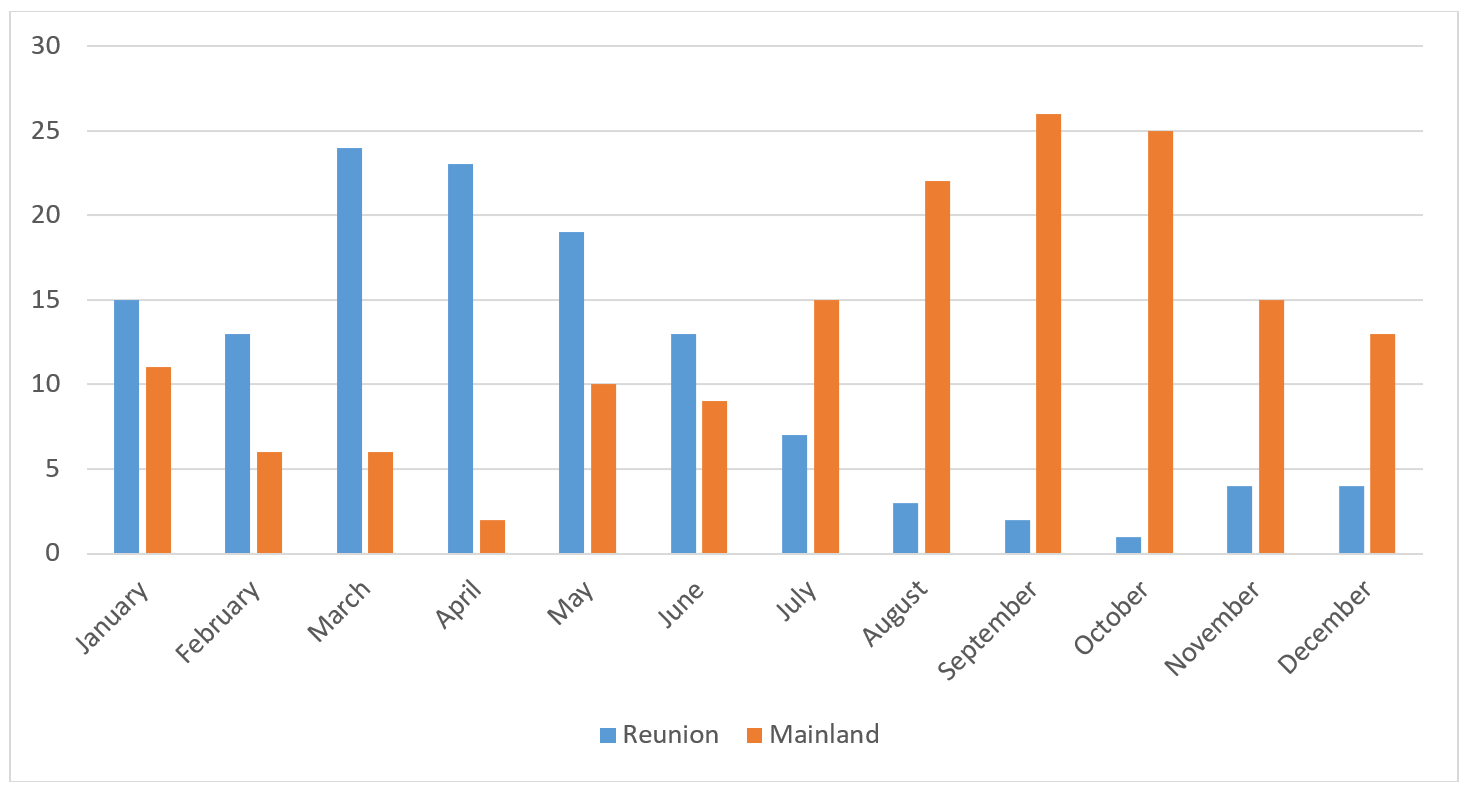

Supplement: S1 Fig — Number of cases of severe leptospirosis, per month, in both cohorts. (TIF) [file pntd.0012084.s002.tif]

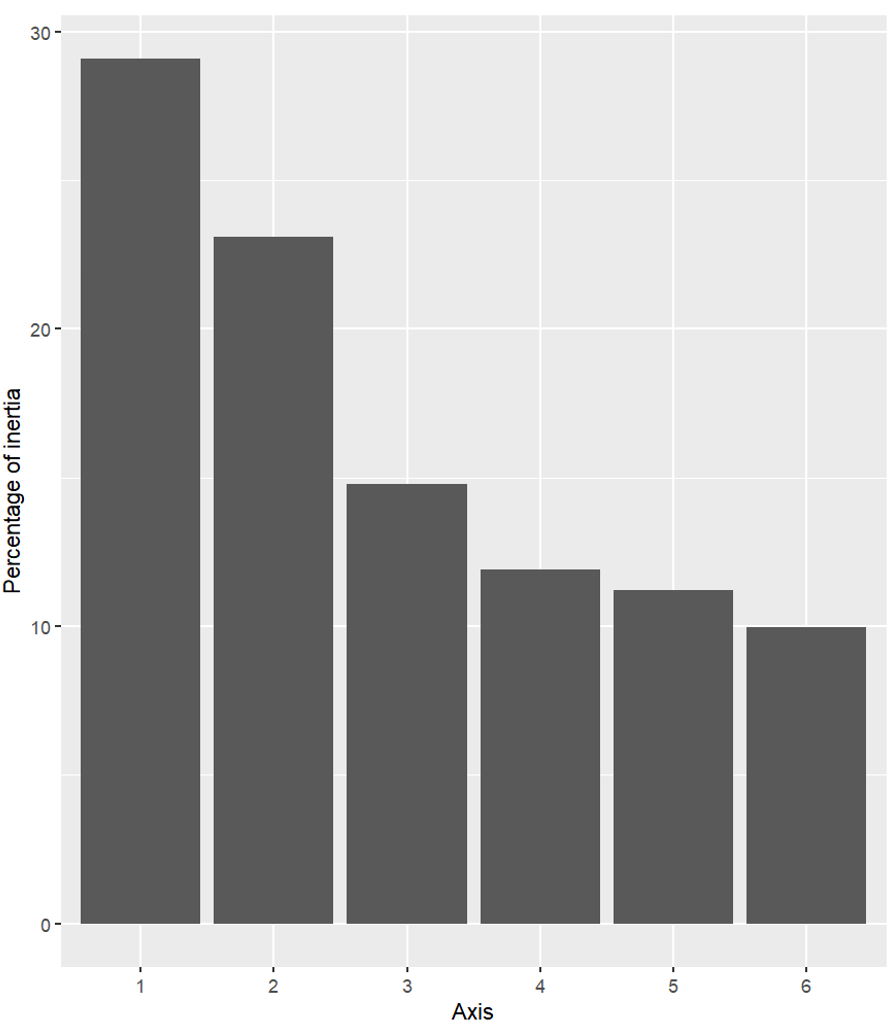

Supplement: S2 Fig — Two patients from Réunion were excluded from the clinical-phenotype analysis due to missing data on organ failure as defined by the SOFA sub-scores. This left 286 patients for the analysis. By multiple correspondence analysis, the cumulative inertia was 66.9% for the first three dimensions. Inertia was 29.1% for the first dimension and 23.1% for the second dimension, yielding a cumulative inertia of 52.2% for the first two dimensions. (TIF) [file pntd.0012084.s003.tif]

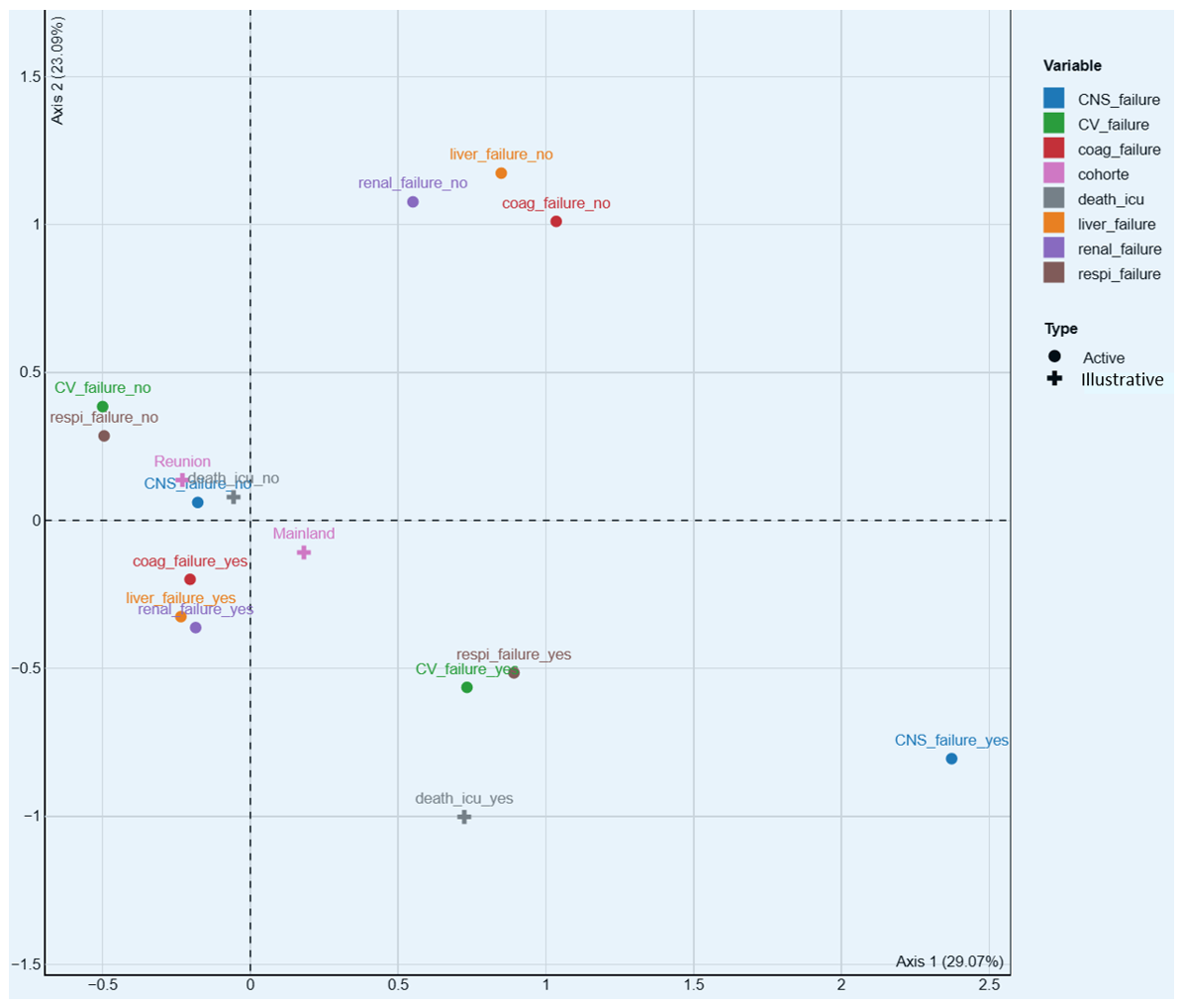

Supplement: S3 Fig — In the first dimension, central nervous system failure, cardiovascular failure, and respiratory failure (righthand side of the graph) are diagonally opposite absence of these events (lefthand side of the graph). Note the close proximity of the two cohorts. CNS: central nervous system; CV: cardiovascular; coag: coagulation; respi: respiratory (TIF) [file pntd.0012084.s004.tif]

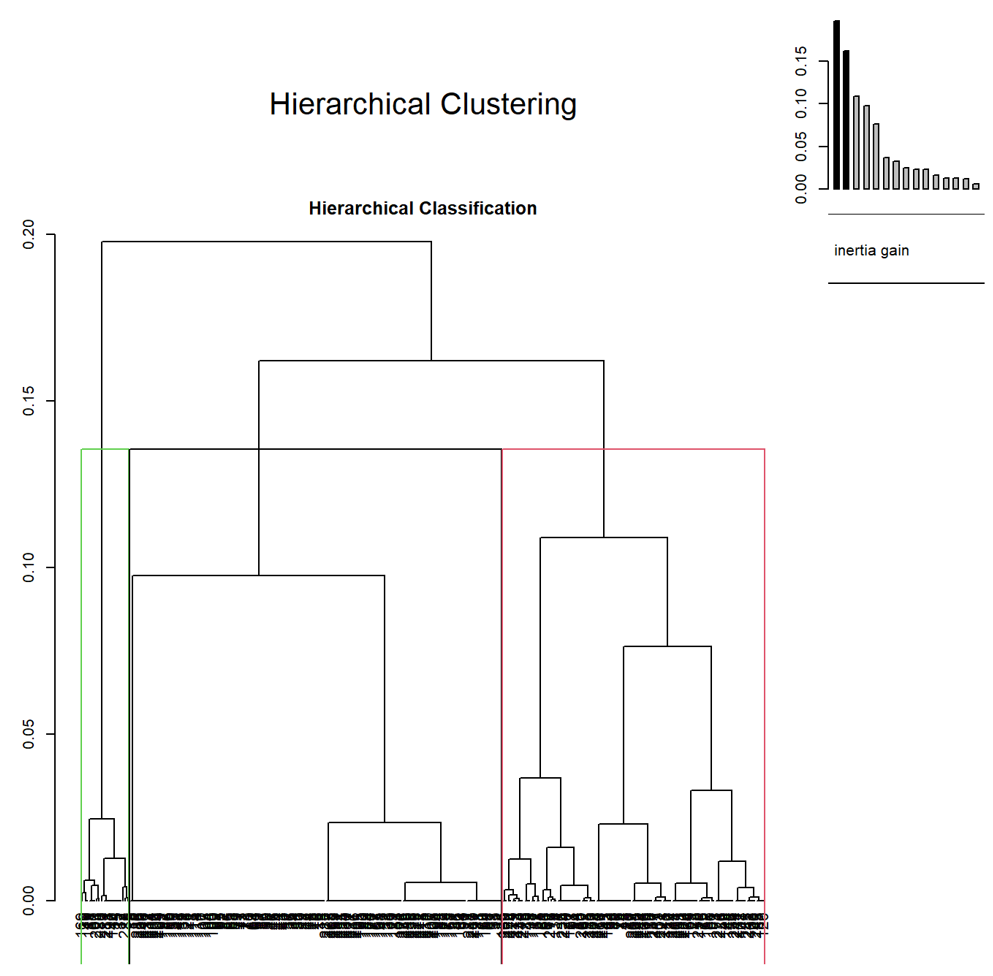

Supplement: S4 Fig — A dendrogram is a tree diagram that illustrates the arrangement of the clusters. Here, individuals are plotted on the X axis. The green, black, and red rectangles define the three clusters of patients: hepato-renal leptospirosis, moderately severe leptospirosis, and neurological leptospirosis, respectively. The graph in the upper right corner represents the loss of inertia along the different dimensions. (TIF) [file pntd.0012084.s005.tif]
