# Supplementary material for: Severe leptospirosis in tropical and non-tropical areas: A comparison of two french, multicentre, retrospective cohorts
Source: PLoS Negl Trop Dis. 2024 Apr 10;18(4):e0012084. doi: 10.1371/journal.pntd.0012084 (PMC11034666; doi:10.1371/journal.pntd.0012084)
Supplement: S1 Acknowledgments — (DOCX) [file pntd.0012084.s006.docx]

**List of contributors :**

Djillali Annane, Service de Médecine Intensive Réanimation, Hôpital Raymond Poincaré (APHP), Faculté Simone Veil des Sciences de la Santé, Université de Versailles Saint-Quentin en Yvelines, Université Paris Saclay, Garches, France

Kevin Arandel, Service de Réanimation Polyvalente, Centre Hospitalier de Cahors, Cahors, France

Pierre Asfar, MD, Service de Médecine Intensive Réanimation, Centre Hospitalier Universitaire Angers, Angers, France

Laurent Argaud, Service de Réanimation médicale, Hôpital Edouard Herriot, Hospices Civils de Lyon, Lyon, France

Antoine Ausseur, Service de Réanimation polyvalente, Centre Hospitalier de Cholet, Cholet, France

Omar Ben Hadj Salem, MD; Réanimation Médico-chirurgicale, Centre Hospitalier Intercommunal de Poissy - Saint-Germain-en-Laye, Poissy, France

Adel Ben Salah, Service de Réanimation Polyvalente, Centre Hospitalier de Chartres, Chartres, France

Pierre-Marie Bertrand, Service de Médecine Intensive Réanimation, CH de Cannes, Cannes, France

Vlad Botoc, Service de Réanimation et Surveillance Continue, Centre Hospitalier de Saint Malo, Saint-Malo, France

Bouju P, Service de Réanimation Polyvalente, CH Bretagne Sud, Lorient, France

Karim Chaoui, Service de Réanimation Polyvalente, Centre Hospitalier de Cahors, Cahors, France

Julien Charpentier, Service de Médecine Intensive Réanimation, Hôpital Cochin, Groupe Hospitalier Centre-Université de Paris, Assistance Publique-Hôpitaux de Paris, Paris, France

Yves Cohen, Service de Réanimation Médico-Chirurgicale, CHU Avicenne, Hôpitaux Universitaires Paris Seine Saint Denis, Assistance Publique-Hôpitaux de Paris, Bobigny, France

Christophe Cracco, Service de Réanimation Polyvalente et Surveillance Continue, Centre Hospitalier d’Angoulême, Angoulême, France

Nicolas De Prost, Service de Réanimation Médicale, Hôpitaux Universitaires Henri Mondor-Albert Chenevier, Assistance Publique-Hôpitaux de Paris, Créteil, France

Roland De Varax, CH de Macon, Service de Médecine Intensive Réanimation, Macon, France

Jean Dellamonica, Service de Médecine Intensive Réanimation, Archet 1, CHU de Nice, Nice, France; Université Côte d’Azur UCA, Nice, France

Jérôme Devaquet, Service de Réanimation Polyvalente, Hôpital Foch, Suresnes, France

Jean-Luc Diehl, Service de Médecine Intensive Réanimation, Hôpital Européen Georges-Pompidou, Assistance Publique-Hôpitaux de Paris, Paris, France

Michel Djibre, Service de Réanimation Medico-Chirurgicale, CHU Tenon, Assistance Publique-Hôpitaux de Paris, Paris, France

Olivier Ellrodt, Service de Médecine Intensive Réanimation, CH Melun, Melun, France

Marie-Line Eustache, Service de Réanimation Polyvalente, Centre Hospitalier Bretagne-Atlantique, Vannes, France

Henri Faure, Service de Réanimation, CH d’Aulnay, Aulnay, France

Alexis Ferré, Service de Réanimation Médico-Chirurgicale, Centre Hospitalier de Versailles, site André Mignot, Le Chesnay, France

Nicolas Ferrière, Service de Réanimation médicale , CHU Brest, Brest, France

Vincent Das, Centre Hospitalier Intercommunal André Grégoire, Réanimation Polyvalente, Montreuil, France

Jacques Durand Gasselin, Service de Réanimation Polyvalente, Centre Hospitalier Sainte-Musse, Toulon, France

Elena Gauvin, Service de Réanimation Polyvalente, Centre Hospitalier de Niort, Niort, France

Suzanne Goursaud, Service de Réanimation Médicale, Centre Hospitalier Universitaire de Caen, Caen, France

Maximilien Grall, Service de Réanimation Médicale, Hôpital Charles Nicolle, Centre Hospitalier Universitaire de Rouen, Rouen, France

Claude Guérin, Service de Réanimation Médicale, CHU Lyon 2, Lyon, France

Bertrand Guidet, Service de Médecine Intensive Réanimation, CHU Saint-Antoine, Assistance Publique-Hôpitaux de Paris, Paris, France

Philippe Guiot, Service de Réanimation Médicale, GHRMSA, Mulhouse, France

Florent Joly, Service de Médecine Intensive Réanimation, CHU Poitiers, Université de Poitiers, Poitiers, France

Maud Jonas, Service de Réanimation Polyvalente et USC, Centre Hospitalier de Saint Nazaire, Saint Nazaire, France

Toufik Kamel, Service de Médecine Intensive Réanimation, CHR Orléans, Orléans, France

Kada Klouche, Service de Médecine Intensive Réanimation, Hôpital Lapeyronnie, CHU de Montpellier, Montpellier, France

Jean-Claude Lacherade, Service de Médecine Intensive Réanimation, Centre Hospitalier Départemental de la Vendée, La Roche-sur-Yon, France

Fabien Lambiotte, Service de Réanimation Polyvalente, Centre Hospitalier de Valenciennes, Valenciennes, France

Mickael Landais, Service de Réanimation Polyvalente, Centre Hospitalier du Mans, Le Mans, France

Jean-Baptiste Lascarrou, Service de Médecine Intensive Réanimation, Centre Hospitalier Universitaire de Nantes, Nantes, France

Guy Le Gall, Service de Médecine Intensive Réanimation, CH Morlaix, Morlaix, France

Jérémie Lemarié, INSERM, U1116, 54500, Vandoeuvre-lès-Nancy, France; Université de Lorraine, Nancy, France; Service de Réanimation Médicale, Centre Hospitalier Universitaire de Nancy, Hôpital Central, Nancy, France

Olivier Lesieur, Service de Réanimation et Surveillance Continue, Hôpital Saint-Louis, La Rochelle, France

Philippe Letocart, Service de Réanimation Polyvalente, Hôpital Jacques Puel, Rodez, France

Claire Lhommet, Service de Réanimation Polyvalente, Centre Hospitalier de Saint Brieuc, Saint Brieuc, France

Charles-Edouard Luyt, Service de Médecine Intensive Réanimation, Institut de Cardiologie, Groupe Hospitalier Pitié-Salpêtrière, Assistance Publique-Hôpitaux de Paris, Paris, France; Université de la Sorbonne, UPMC Université Paris 06, INSERM, UMRS_1166-ICAN Institute of Cardiometabolism and Nutrition, Paris, France

Adel Maamar, Service de Médecine Intensive Réanimation, Centre Hospitalier Universitaire de Rennes, Rennes, France

Philippe Mateu. Service de Médecine Intensive Réanimation, CH Charleville-Mézière, Charlesville-Mézière, France

Emmanuelle Mercier, Service de Médecine Intensive Réanimation, Centre Hospitalier Universitaire Bretonneau, CRICS-TRIGGERSEP network Tours, France

Hamid Merdji, Université de Strasbourg (UNISTRA), Faculté de Médecine, Hôpitaux Universitaires de Strasbourg, Service de Réanimation, Nouvel Hôpital Civil, Strasbourg, France

Olivier Michel, Service de Réanimation, Centre Hospitalier Jacques Coeur, Bourges, France

Philippe Michel, Service de Réanimation Médico-Chirurgicale, Centre Hospitalier René-Dubos, Pontoise, France

Yannick Monseau, Service de Réanimation Polyvalente, Centre Hospitalier de Périgueux, Périgueux, France

Sébastien Moschietto, Service de Médecine Intensive Réanimation, Centre Hospitalier Henri Duffaut, Avignon, France

Saad Nseir, Médecine Intensive Réanimation, Centre Hospitalier Universitaire de Lille, Lille, France; Université de Lille, Faculté de Médecine, Lille, France

David Osman, Service de Médecine Intensive Réanimation, Centre Hospitalier Universitaire Bicêtre, Assistance Publique-Hôpitaux de Paris, Paris, France

Laurent Papazian, Centre d'Etudes et de Recherches sur les Services de Santé et Qualité de Vie EA3279, Assistance Publique-Hôpitaux de Marseille, Hôpital Nord, Médecine Intensive Réanimation, Aix-Marseille Université, 13015, Marseille, France

Olivier Passouant, Service de Réanimation Polyvalente, Hôpital Robert Debré, CHU de Reims, Reims, France

Walter Picard, Service de Réanimation, CH de Pau, Pau, France

Jérome Pillot, Service de Réanimation Polyvalente, Hôpital Saint-Léon, Centre Hospitalier de la Côte Basque, Bayonne, France

Gaël Piton, Service de Médecine Intensive Réanimation, Centre Hospitalier Universitaire de Besançon, Besançon, France

Fabienne Plouvier, Service de Réanimation Polyvalente, CH Agen, Agen, France

Jean-Pierre Quenot, Service de Médecine Intensive Réanimation, CHU François Mitterrand, Dijon, France; Lipness Team, INSERM LNC-UMR1231, LabExLipSTIC, INSERM CIC 1432, Epidémiologie Clinique, Université de Bourgogne, Dijon, France

Jean-Damien Ricard, INSERM, IAME, UMR 1137, F-75018 Paris, France; Service de Réanimation Médico-Chirurgicale, Hôpital Louis Mourier, Assistance Publique-Hôpitaux de Paris, Colombes, France; Université Paris Diderot, IAME, UMR 1137, Sorbonne Paris Cité, Paris, France

Jean-Philippe Rigaud, Service de Médecine Intensive Réanimation, CH Dieppe, Dieppe, France

Jérôme Roustan, Service de Médecine Intensive Réanimation, Centre Hospitalier de Montauban, Montauban, France

Frédérique Schortgen, Service de Réanimation et de Surveillance Continue, Centre Hospital Intercommunal, Créteil, France

Nicholas Sedillot, Service de Réanimation Polyvalente, Centre Hospitalier Fleyriat, Bourg-en-Bresse, France

Michel Sirodot, Service de Médecine Intensive Réanimation, Centre Hospitalier Annecy, Annecy, France

Bertrand Souweine, Service de Médecine Intensive Réanimation, CHU Clermont-Ferrand, Clermont-Ferrand, France

Benjamin Sztrymf, Service de Médecine Intensive Réanimation, Hôpital Antoine Béclère, Assistance Publique-Hôpitaux de Paris, Clamart, France; INSERM U999 "HTAP: Physiopathologie et innovation thérapeutique

Nicolas Terzi, Service de Médecine Intensive Réanimation, CHU de Grenoble, Grenoble, France; Inserm U 1032, Université de Grenoble, Grenoble, France

Didier Thevenin, Service de Médecine Intensive Réanimation, Centre Hospitalier de Lens, Lens, France

Thierry Vanderlinden, Service de Médecine Intensive Réanimation, GH ICL, Lomme, France

Philippe Vignon, Service de Reanimation, CHU de Limoges, Limoges, France

Christophe Vinsonneau, Service de Réanimation et Surveillance Continue, Centre Hospitalier de Béthune, Béthune, France

Lara Zafrani, Service de Médecine Intensive Réanimation, Centre Hospitalier Universitaire Saint Louis, Assistance Publique-Hôpitaux de Paris, Paris, France

Fabrice Zeni, Service de Médecine Intensive Réanimation, CHU de Saint Etienne, Saint Etienne, France

Yoann Zerbib, Service de Réanimation médicale, Centre Hospitalier Universitaire Amiens-Picardie, Amiens, France
